# Reduced glucose concentration enhances ultradian rhythms in *Pdcd5* promoter activity *in vitro*

**DOI:** 10.3389/fphys.2023.1244497

**Published:** 2023-10-11

**Authors:** Isaiah J. Ting, Andreas Psomas, Debra J. Skene, Daan R. Van der Veen

**Affiliations:** Chronobiology Section, Faculty of Health and Medical Sciences, University of Surrey, Guildford, United Kingdom

**Keywords:** ultradian, circadian, co-expression, adipose, metabolism, PDCD5, glucose, hypoglycemia

## Abstract

Intrinsically driven ultradian rhythms in the hourly range are often co-expressed with circadian rhythms in various physiological processes including metabolic processes such as feeding behaviour, gene expression and cellular metabolism. Several behavioural observations show that reduced energy intake or increased energy expenditure leads to a re-balancing of ultradian and circadian timing, favouring ultradian feeding and activity patterns when energy availability is limited. This suggests a close link between ultradian rhythmicity and metabolic homeostasis, but we currently lack models to test this hypothesis at a cellular level. We therefore transduced 3T3-L1 pre-adipocyte cells with a reporter construct that drives a destabilised luciferase via the *Pdcd5* promotor, a gene we previously showed to exhibit robust ultradian rhythms *in vitro*. Ultradian rhythmicity in *Pdcd5* promotor driven bioluminescence was observed in >80% of all cultures that were synchronised with dexamethasone, whereas significantly lower numbers exhibited ultradian rhythmicity in non-synchronised cultures (∼11%). Cosine fits to ultradian bioluminescence rhythms in cells cultured and measured in low glucose concentrations (2 mM and 5 mM), exhibited significantly higher amplitudes than all other cultures, and a shorter period (6.9 h vs. 8.2 h, N = 12). Our findings show substantial ultradian rhythmicity in *Pdcd5* promotor activity in cells in which the circadian clocks have been synchronised *in vitro*, which is in line with observations of circadian synchronisation of behavioural ultradian rhythms. Critically, we show that the amplitude of ultradian rhythms is enhanced in low glucose conditions, suggesting that low energy availability enhances ultradian rhythmicity at the cellular level *in vitro*.

## 1 Introduction

Ultradian rhythms are rapidly cycling biological rhythms, which are expressed with periods ranging from milliseconds to hours in physiology and behaviour in a plethora of species, extending from yeast to humans. Ultradian rhythms are often co-expressed with circadian (∼24 h) rhythms, which was first reported by Szymansky when looking at behavioural activity patterns in a range of species (Szymanski, 1920). Since then, ultradian rhythms have been reported in numerous behavioural and physiological processes such as locomotor and feeding behaviour (Gerkema and van der Leest, 1991; van Oort et al., 2007; Dowse et al., 2010; van Beest et al., 2020; Morris et al., 2022), body temperature (Lindsley et al., 1999; Diatroptov et al., 2019; 2020; Thiel et al., 2022), glucocorticoid concentration (Lightman et al., 2008; Waite et al., 2012; Flynn et al., 2018), hormone release (Van Cauter, 1990; Droste et al., 2008; Waite et al., 2012; Kalafatakis et al., 2018), central monoamine release (Blum et al., 2014), gene expression (Hughes et al., 2009; van der Veen and Gerkema, 2017; Ballance and Zhu, 2021; Scott et al., 2023), and cellular metabolism (Brodsky, 2014; Zhu et al., 2017; Goh et al., 2019; Ballance and Zhu, 2021; Yang et al., 2022; Asher and Zhu, 2023; Psomas et al., 2023). Despite these widespread observations of ultradian rhythms, very little is known about their function and the intrinsic mechanisms that drive them.

Ultradian dynamics in feeding behaviour and metabolic physiology can be externally imposed (or masked) by e.g., polyphasic feeding activity. However, it is becoming increasingly clear that ultradian rhythms in processes such as behaviour, endocrine activity, gene expression and cellular metabolism can also be intrinsically driven (Gerkema and van der Leest, 1991; van der Veen et al., 2011; Bloch et al., 2013; Blum et al., 2014; van der Veen and Gerkema, 2017; Flynn et al., 2018; Wu et al., 2018; Aviram et al., 2021; Yang et al., 2022; Psomas et al., 2023). The mechanisms that drive intrinsic behavioural ultradian rhythms have been shown to involve specific brain regions such as the retrochiasmatic nucleus (RCA) and dopamine oscillations in the midbrain (Gerkema and Daan, 1990; Blum et al., 2014), but an ultradian oscillator has not yet been identified. Importantly, whilst often co-expressed with circadian rhythms, intrinsic ultradian behavioural rhythms are independent from circadian clocks, and persist when circadian clocks are removed, either through lesioning of the suprachiasmatic nuclei (SCN) of the hypothalamus (Gerkema and Daan, 1990; Schwartz and Zimmerman, 1991; Prendergast et al., 2012; Prendergast and Zucker, 2016; Goh et al., 2019) or through ablation of circadian clock components in mammals (Vitaterna et al., 1994; Bunger et al., 2000; Zheng et al., 2001; Aviram et al., 2021; Ballance and Zhu, 2021).

The independence of ultradian rhythms in behavioural observations is particularly highlighted during periods of reduced energy intake or increased energy expenditure in which the relative contribution of ultradian rhythms increases at the expense of circadian timing (Gerkema and van der Leest, 1991; van der Veen et al., 2006; van Oort et al., 2007; van Beest et al., 2020; van Rosmalen and Hut, 2021). The mechanism that underlies this striking prioritisation of ultradian timing over circadian timing when energy availability is reduced is not well understood but alludes to the early hypotheses of a relationship between ultradian rhythms and metabolic homeostasis (Aschoff and Meyer-Lohmann, 1954; Aschoff, 1960; Aschoff and Gerkema, 1985). In support of this hypothesis are the recent observations that ultradian rhythms are particularly evident in expression of genes linked to metabolism (Enright, 1989; Hughes et al., 2009; van der Veen and Gerkema, 2017). Ultradian rhythms have also been identified at the cellular level in metabolic processes such as protein synthesis, intracellular ATP concentration, cell respiration, and glutaminolysis (Brodsky, 2014; Aviram et al., 2021; Ghenim et al., 2021; Yang et al., 2022). Taken together, these findings strongly suggest that ultradian rhythmicity is linked to metabolic homeostasis at a cellular level, and that the expression of ultradian rhythms is enhanced in times when energy availability is limited. This hypothesis, however, is based only on observations, and we currently lack *in vitro* models to test this at a cellular level.

Here, we have developed an *in vitro* model of ultradian rhythmicity in which *Pdcd5* (Programmed Cell Death 5) expression activity (van der Veen and Gerkema, 2017) is reported in real-time using bioluminescence expression in pre-adipocyte cells. *Pdcd5* is a gene involved in programmed cell death which exhibits robust co-expression of ultradian rhythms in NIH 3T3 cells *in vitro* (van der Veen and Gerkema, 2017). Using this model, we show that reducing energy availability *in vitro* enhances ultradian rhythmicity in *Pdcd5* promotor activity at the cellular level.

## 2 Materials and methods

All cell culture work was conducted in a type 2 laminar flow cabinet (ThermoFisher Scientific, 41940037) and all solutions were pre-warmed in a water bath (Isotemp^©^; ThermoFisher Scientific, 300232999) at 37°C. Cultured cells were incubated at 37°C in a humidified atmosphere with 5% carbon dioxide (CO_2_) in a CellXpert incubator (Eppendorf, 6734I6002000).

### 2.1 Experimental protocol

**FIGURE 1:**
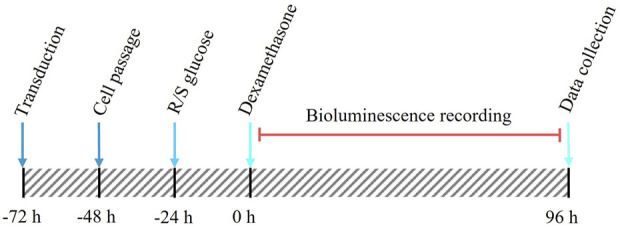
Schematic timeline of the cell culture protocol from transduction to data collection. Red line indicates the bioluminescence recording period (96 h).

**FIGURE 2:**
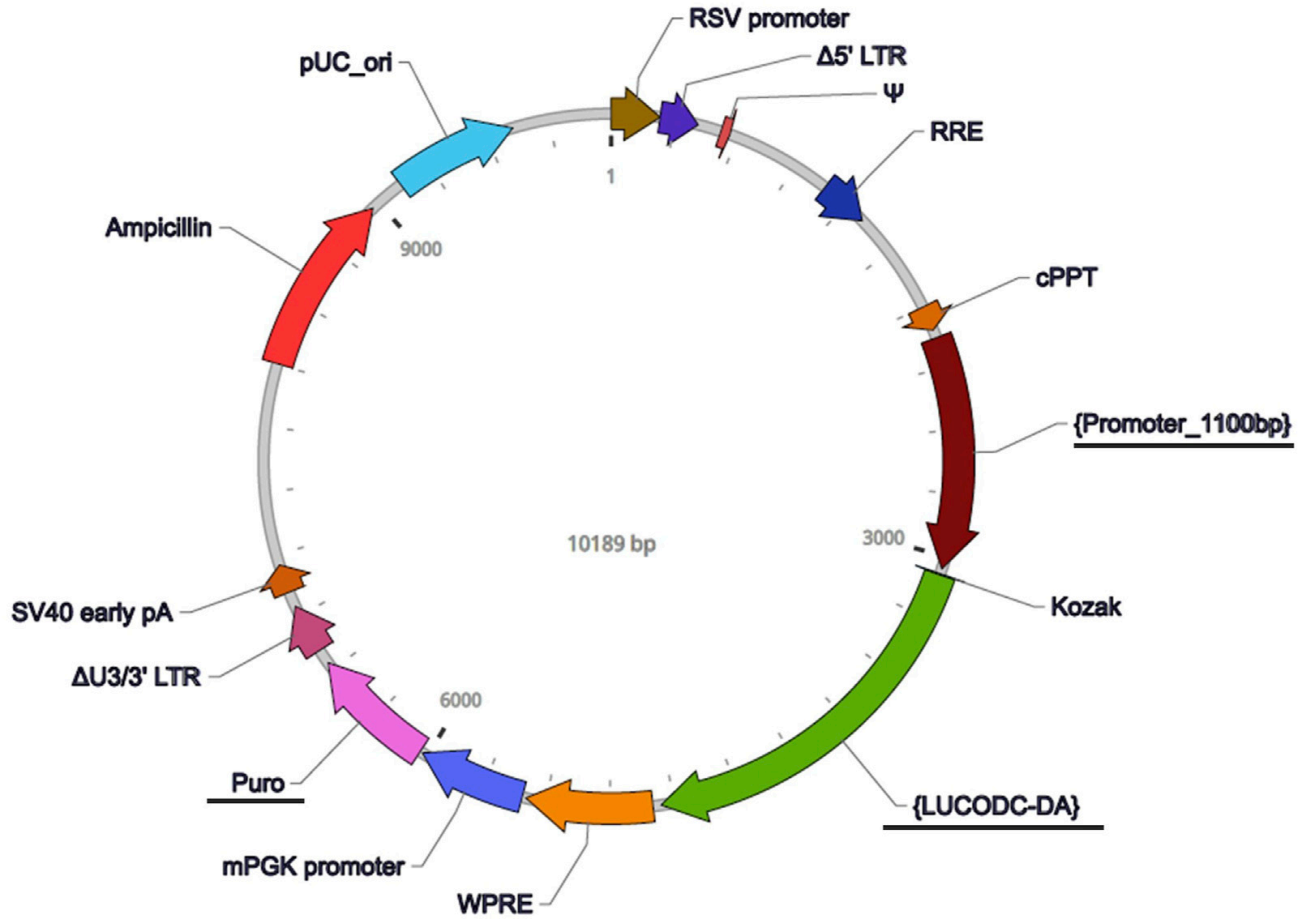
Vector map for the *Pdcd5* reporter construct. The lentiviral vector used to track promoter activity of *Pdcd5* was constructed and packaged by VectorBuilder. Vector components include RSV promoter (Rous sarcoma virus promoter), Δ5′ LTR (Truncated HIV-1 5′ long terminal repeat), Ψ (HIV-1 packaging signal), RRE (HIV-1 Rev response element), cPPT (Central polypurine tract), {Promoter_1100bp} (*Pdcd5* promoter), Kozak (Kozak translation initiation sequence), {LUCODC-DA} (destabilised luciferase sequence), WPRE (Woodchuck hepatitis virus posttranscriptional regulatory element), mPGK promoter (Mouse phosphoglycerate kinase-1 promoter), Puro (Puromycin resistance gene), ΔU3/3′ LTR (Truncated HIV-1 3′ long terminal repeat), SV40 early pA (Simian virus 40 early polyadenylation signal), Ampicillin (Ampicillin resistance gene), and pUC_ori (pUC origin of replication). Features that are underlined were components that have been custom added to detect *Pdcd5* expression by fusing the *Pdcd5* promoter containing area to a destabilised luciferase.

The experimental approach is shown in Figure 1, with experimental groupings in Table 1. In short, pre-adipocyte cells were transduced with a custom-made destabilised luciferase reporter construct (Figure 2) and transferred to 35 mm dishes. These cultures were then incubated with media containing either standard glucose concentration (50 mM) or reduced glucose concentrations (2, 5 or 10 mM) for 24 h prior to synchronisation of the circadian clocks with dexamethasone for 30 min. After synchronisation, cells were cultured in media containing either standard glucose concentration (for S/S and R/S conditions) or media containing reduced glucose concentrations (for R/R condition; Table 1). Cultures were then placed in the LumiCycle for a total of 4 days (96 h) to measure *Pdcd5* promoter-driven bioluminescence. All conditions, including the negative controls, were performed to a biological replicate of 12, and each biological replicate comprised of 3 technical replicates. Rhythmicity in each of the technical replicates was recorded and for a biological replicate to be considered “rhythmic”, ≥2 out of 3 technical replicates must be rhythmic.

**TABLE 1:** Experimental controls and conditions.

	Transduced	Luciferin	Synchronised	Glucose concentration during culturing				Glucose concentration during measurement			
				2 nM	5 nM	10 nM	50 nM	2 nM	5 nM	10 nM	50 nM
Non-transduced (**NT**)	—	+	+	—	—	—	+				+
Non-luciferin (**NL**)	+	—	+	—	—	—	+	—	—	—	+
Non-synchronised (**NS**)	+	+	—	—	—	—	+	—	—	—	+
Standard glucose (**S/S**)	+	+	+	—	—	—	+	—	—	—	+
Reduced glucose: **R2/S**	+	+	+	+	—	—	—	—	—	—	+
Reduced glucose: **R5/S**	+	+	+	—	+	—	—	—	—	—	+
Reduced glucose: **R10/S**	+	+	+	—	—	+	—	—	—	—	+
Reduced glucose: **R/R2**	+	+	+	+	—	—	—	+	—	—	—
Reduced glucose: **R/R5**	+	+	+	—	+	—	—	—	+	—	—
Reduced glucose: **R/R10**	+	+	+	—	—	+	—	—	—	+	—

### 2.2 *Pdcd5::dLUC* reporter construct

To track transcriptional activity of the *Pdcd5* gene *in vitro*, we designed a reporter construct that contained a copy of the murine *Pdcd5* promotor area (defined as the 1100 bp DNA sequence upstream of the protein translation start) fused to a destabilised luciferase (LUCODC-DA; functional half-life of 0.84 h; Leclerc GM et al., 2000). To note, the UCSC genome browser indicates 2 transcription start sites for the *Pdcd5* gene in the mouse, one of which is present within our reporter construct for *Pdcd5* (UCSC ID: *uc009gkf.2*). The reporter was constructed and packaged commercially by VectorBuilder, Texas, United States (Figure 2). Permanent integration of the reporter construct was achieved by transducing murine cells using a third generation Lentivirus Gene Expression Vector containing the reporter construct (Dull et al., 1998).

### 2.3 Pre-adipocyte cell culturing and infection

Murine pre-adipocyte cells (3T3-L1) were purchased from Merck (product #93061524), at a density of 1 × 10^6^ cells/mL per vial. Cells were cultured in Dulbecco’s Modified Eagle’s Medium (DMEM; Sigma-Aldrich, D6546) containing 4,500 mg/L glucose, sodium bicarbonate and sodium pyruvate. DMEM was supplemented with 10% Calf serum (CS; Sigma-Aldrich, C8056), 2 mM L-glutamine (Sigma-Aldrich, G7513) and 1% antibiotic-antimycotic (100 units/mL; ThermoFisher Scientific, 2211104).

Pre-adipocyte cells were grown in T75 culture flasks to reach 70%–90% confluency. For passaging, the culture medium was aspirated, and cells were washed with pre-warmed 1 x phosphate buffered saline twice (PBS; Sigma- Aldrich, D8537). For cell detachment, 5 mL of 1 x Trypsin-EDTA (Sigma-Aldrich, T3924) was added to the cells before incubation at 37°C, 5% CO_2_, for 4 min. To ensure full detachment of cells, flasks were gently tapped and checked under a microscope for confirmation of full cell detachment. Trypsin-EDTA was then inactivated by addition of 5 mL of fresh culture media to the flask. The cell suspension was transferred to a 15 mL falcon tube and then centrifuged at 600 *g* for 10 min, at 4°C. After centrifugation, the supernatant was removed, and the cell pellet was resuspended in 2 mL of fresh culture medium. The cell suspension was either placed back in culture flasks for further growth or placed in appropriate media and dishes for further experiments.

For transduction, pre-adipocyte cells (passage 3–4) were seeded and grown to 50%–60% confluency in 35 mm dishes. On reaching the desired confluence, each 35 mm dish of pre-adipocyte cells was infected with the lentiviral particles, with a multiplicity of infection (MOI) of 2, by dissolving 2 × 10^13^ viral particles in 2 mL DMEM, supplemented with 50 mM glucose, 10% calf serum, 2 mM L-glutamine, 1% antibiotic-antimycotic and 8 ug/mL of protamine sulphate (Sigma-Aldrich, P4020). After 24 h of exposure to the transduction medium, cells were washed with pre-warmed PBS and then transferred to T75 culture flasks or 35 mm dishes for further experiments.

### 2.4 Circadian clock synchronisation

Cells were treated with the synthetic glucocorticoid dexamethasone (DEX, Sigma-Aldrich) to synchronise the phase of the circadian oscillators between pre-adipocyte cells (Balsalobre et al., 1998; Balsalobre et al., 2000). Once the pre-adipocyte cells reached 60%–70% confluence in 35 mm dishes, cells were synchronised using dexamethasone (DEX) at a concentration of 0.1 µM in 2 mL of culture medium and incubated for 30 min (Bieler et al., 2014). At the end of the incubation, the DMEM-DEX solution was aspirated, the dishes were washed with pre-warmed PBS and fresh culture medium was added to the cells. The start of DEX synchronisation is taken as timepoint 0.

### 2.5 Bioluminescence recording of *Pdcd5* promoter activity

We recorded *Pdcd5* promoter-driven bioluminescence for 4 days (96 h) using a LumiCycle 32-channel luminometer (Actimetrics) with pre-adipocyte cells at passage 3–4 (passage 1 represents cells passaged after purchasing from supplier) transduced with the *Pdcd5*::d*Luc* reporter construct. Transduced and non-transduced pre-adipocyte cells were plated at a density of 1 × 10^5^ cells/mL in 35 mm dishes containing 2 mL of DMEM and were incubated for 24 h at 37°C with 5% CO_2_ and then synchronised. For conditions with specified reduced glucose concentration, DMEM without glucose was used (Thermo Fisher Scientific, A1443001), and supplemented with the same additive concentrations as high glucose DMEM, except for glucose which was added with the desired reduced glucose concentrations. Afterwards, the cells were washed twice with pre-warmed PBS and then treated with 2 mL of bioluminescence solution that contained 10% calf serum, 1% antibiotic-antimycotic, 1% L-Glutamine, and 0.1 mM Luciferin-EF™ (Promega, E6551) in DMEM with the varying levels of glucose concentration (2, 5 or 10 mM) depending on the treatment group. The dishes were sealed with glass coverslips which were secured with silicon grease (ThermoFisher Scientific, 12302058), and then transferred to the LumiCycle for bioluminescence monitoring and recording. *Pdcd5*-driven bioluminescence was recorded every 10 min as photons/second, and after the measurement duration of 4 days, raw data were exported from the Actimetrics LumiCycle software for further analysis.

### 2.6 Data analysis

The first 24 h of the 4-day recording was discarded to remove the immediate effects of dexamethasone supplementation. Bioluminescence recordings were baseline detrended using a 16 h running average to remove circadian and/or other long-term variations, and mean expression levels were compared between treatment groups using a one-way ANOVA. The total number of rhythmic cultures per group were then compared using a Chi-square test, where the number of rhythmic groups in the standard glucose condition (S/S) was taken as the expected values and multiple comparisons between the S/S group as compared to the R/S and R/R groups were performed. Rhythmicity analysis consisted of cosine curve fitting, which was statically tested against a straight line using an extra-sum-of-squares F test (Psomas et al., 2023). Cosinor parameters such as period and amplitude were compared between experimental groups using a 2-way ANOVA (levels were 1) effect of synchronising cultures and 2) different glucose concentrations).

## 3 Results

**FIGURE 3:**
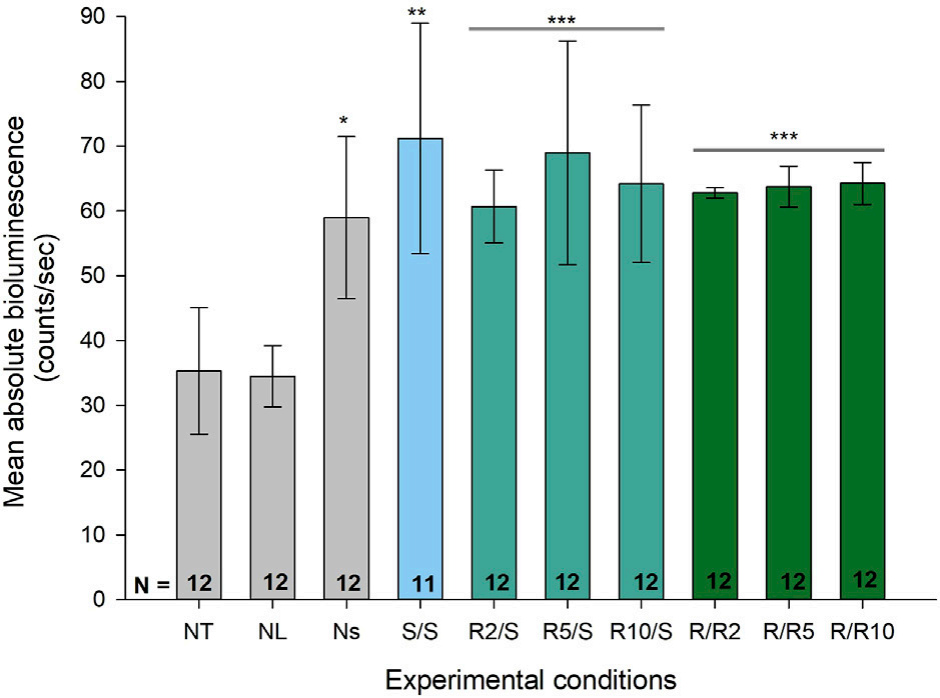
Absolute bioluminescence levels across different experimental conditions including negative controls. Bar graph representing the mean, absolute bioluminescence levels (±SD) across different experimental conditions. Group biological replicates are indicated at the base of the bar, where each biological replicate comprised of 3 technical replicates. *p < 0.05 against negative controls (NT and NL), **p < 0.01 and ***p < 0.001 by one-way ANOVA, and by Tukey’s multiple comparisons test for each condition compared to the negative control.

To test our hypothesis that expression of ultradian rhythmicity at a cellular level is enhanced in conditions of reduced glucose availability, we designed an *in vitro* model assessing ultradian activity of the *Pdcd5* promotor under normal and reduced glucose conditions in pre-adipocytes. We first confirmed that the *Pdcd5::*d*Luc* reporter construct drove bioluminescence *in vitro* by comparing bioluminescence levels between negative control cultures (non-transduced; NT and non-luciferin; NL) to positive control cultures that were transduced and cultured in the presence of luciferin during bioluminescence measurement (non-synchronised; Ns). Mean bioluminescence levels in *Pdcd5::*d*Luc* (N = 12 biological replicates, each comprising 3 technical replicates) over 72 h (days 2–4) was significantly higher in transduced cells (58.9 ± 12.0 counts/sec, Figure 3) compared to negative controls (NT; mean ± SD: 34.5 ± 4.5 counts/sec and NL 35.3 ± 9.4 counts/sec, Tukey’s HSD test, *p* < 0.01). We then confirmed that mean levels of expression in the experimental groups were all significantly higher than the negative controls (S/S; 72.2 ± 17.0 counts/sec; R/S; 64.3 ± 11.8 counts/sec; R/R; 63.6 ± 2.6 counts/sec, Tukey’s HSD test, *p* < 0.001). Within the reduced glucose conditions (R/S and R/R), no significant differences were found in mean bioluminescence levels between glucose concentrations from 2 mM to 10 mM (Tukey’s HSD test, *p* > 0.05) within the R/S and R/R groups.

**FIGURE 4:**
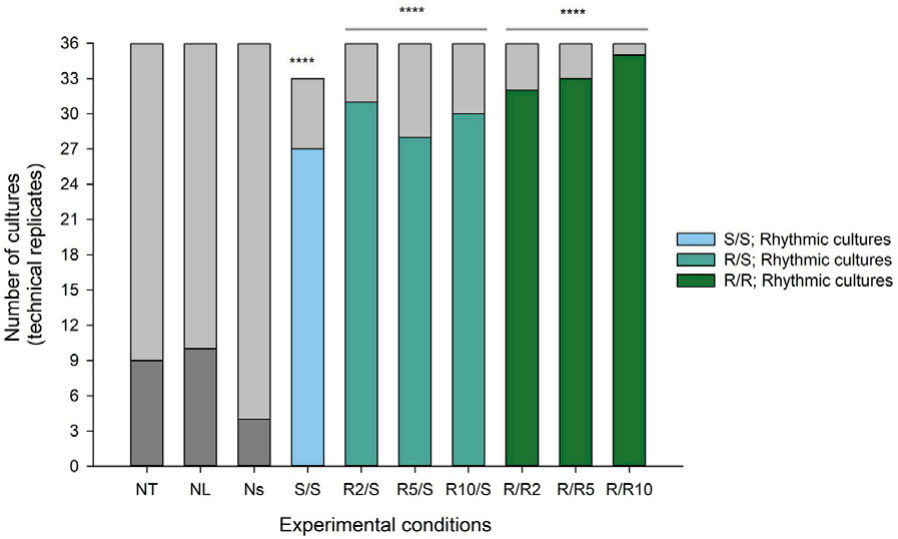
Number of cultures expressing ultradian rhythms in *Pdcd5::*d*Luc* *driven bioluminescence*. Stacked bar graph representing the number of rhythmic (coloured) and non-rhythmic cultures (light grey) in all experimental conditions. A chi-squared test of independence was performed to examine the relationship between reduced glucose conditions and presence of ultradian rhythmicity in cultures. Experimental groups were compared to Ns negative control, and the relationship between variables was significant for all comparisons. ****p < 0.0001 against negative control (Ns).

**FIGURE 5:**
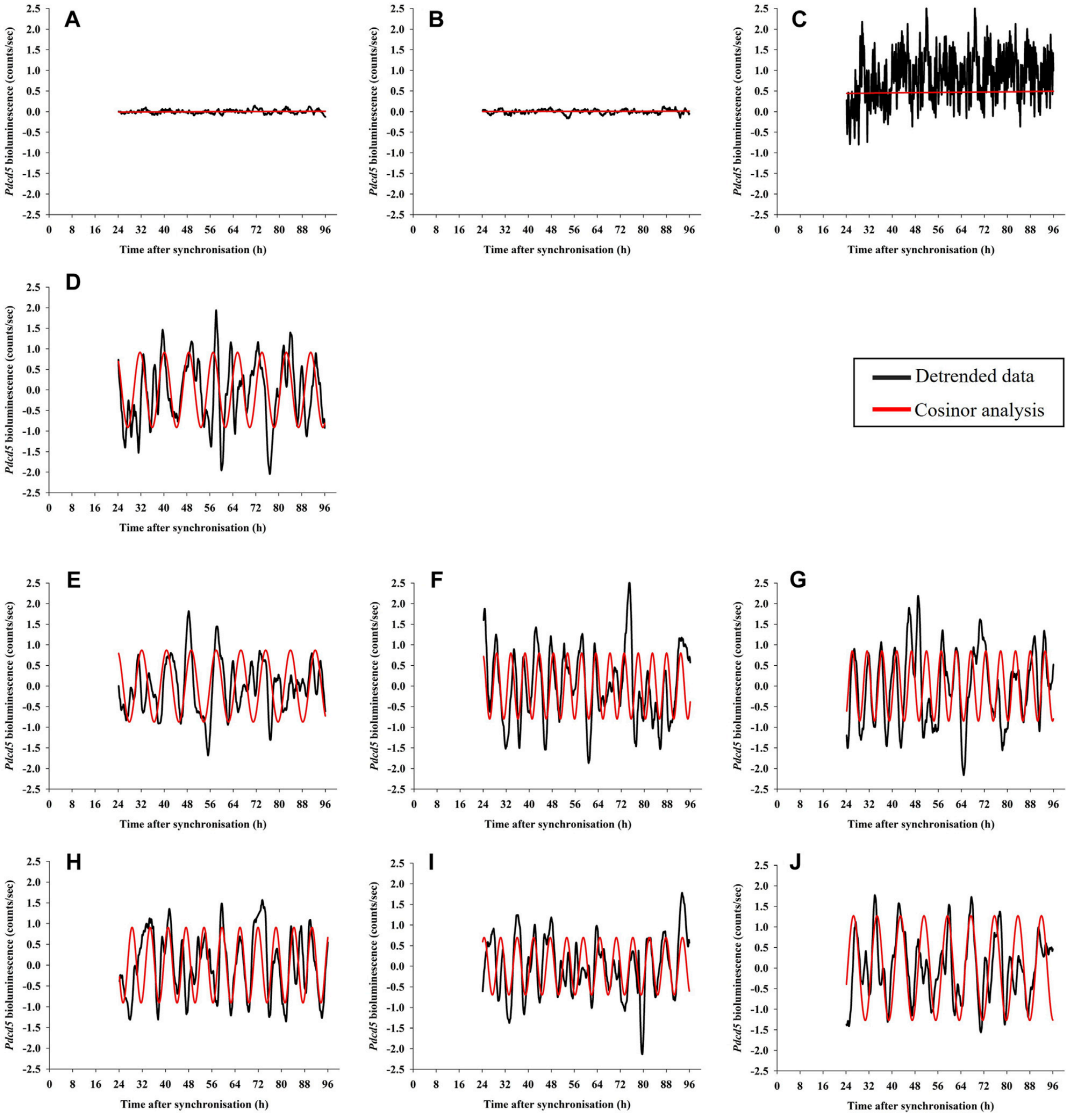
Examples of *Pdcd5::*d*Luc* bioluminescence recordings in pre-adipocyte cells. Detrended data (black line) was analysed for presence of rhythms using cosinor analysis and compared to a straight line (extra-sum-of-squares F-test; red line). The panels shows representative examples of bioluminescence from cultures that are Non-transduced (NT; panel **(A)**, Non-luciferin (NL; panel **(B)**, Non-synchronised (Ns; panel **(C)**, Standard glucose (S/S, 50 mM; panel **(D)**, cultures with reduced glucose only at pre-synchronisation with varying glucose concentrations (R/S, 2 mM, 5 mM and 10 mM; R2/S: panel **(E)**, R5/S: panel **(F)**, R10/S: panel **(G)**), and cultures with reduced glucose both at pre- and post-synchronisation (R/R, 2 mM, 5 mM and 10 mM; R/R2: panel **(H)**, R/R5: panel **(I)**, R/R10: panel **(J)**.

We next characterised the number of cultures (12 biological replicates * 3 technical replicates = 36 cultures per condition) expressing significant ultradian rhythms in *Pdcd5::*d*Luc*-driven bioluminescence as determined by cosinor analysis. Ultradian cosine fitting identified low numbers of ultradian rhythmicity in the negative controls (NT and NL; 9 and 10 rhythmic cultures, respectively), and only 4 rhythmic cultures were found in the non-synchronised (Ns) positive control (Figure 4). Visual inspection (Figures 5A–C) confirmed that the negative controls exhibited very low levels of variation in background bioluminescence over time, whereas the non-synchronised (Ns) controls exhibited high levels of bioluminescence with high-frequency, irregular variation which did not fit a cosine curve. The bioluminescence pattern in cultures that were synchronised and cultured in standard glucose (S/S; 50 mM) exhibited significant ultradian rhythms in 27 out of the 33 (82%) cultures measured (Figure 4), which was characterised by high amplitude, regular rhythms in *Pdcd5* driven bioluminescence (Figure 5D). When looking at the number of rhythmic cultures when glucose concentrations were reduced, either pre-synchronisation only (R/S), or pre- and post-synchronisation (R/R), we observed that on average, 30/36 (83%) cultures in the R/S groups and 33/36 (92%) cultures in the R/R groups were rhythmic.

Chi-square analysis showed that synchronising the cellular circadian clocks using DEX associated with a significantly higher number of ultradian rhythmic cultures (S/S: χ^2^ (1, N = 11) = 36.7, *p* < 0.0001) as well as culturing in reduced glucose concentrations (R/S: χ^2^ (1, N = 12) = 37.7, *p* < 0.0001.; R/R: χ^2^ (1, N = 12) = 46.8, *p* < 0.0001). We observed that when glucose concentrations were reduced at pre- DEX synchronisation, rhythmic cultures made up 31/36 (86%) cultures for R2/S, 28/36 (78%) cultures for R5/S and 30/36 (83%) cultures for R10/S. For cultures that were at reduced glucose concentrations at pre- and post-synchronisation, rhythmic cultures made up 32/36 (89%) cultures for R/R2, 33/36 (92%) cultures for R/R5 and 35/36 (97%) cultures for R/R10 (Figure 4). Comparison of rhythmic cultures within the different reduced glucose concentrations showed no significant differences between conditions (*p* values >0.05, see Supplementary Table S1).

**FIGURE 6:**
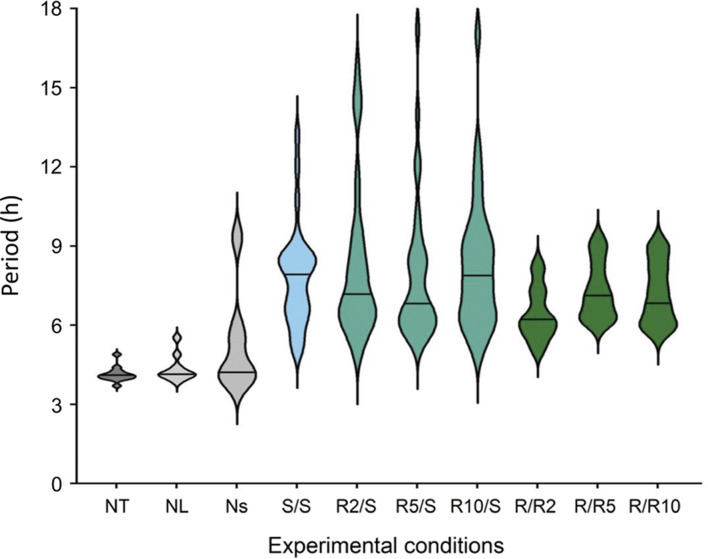
Violin plots representing the period distribution of ultradian rhythms of *Pdcd5::*d*Luc* bioluminescence under different glucose conditions compared with negative controls. Black solid line represents the mean period found across all biological replicates (N = 12) per condition, excluding the S/S condition which represents the mean period across N = 11. ***p < 0.001 by one-way ANOVA, and by Tukey’s multiple comparisons test against negative controls (NT, NL), ^α^ *p* < 0.002 by one-way ANOVA, and by Tukey’s multiple comparisons test against R/S. *See* Supplementary Table S2 *for detailed comparisons.*

Cosine fitting also provided estimates of the periods of the ultradian rhythms expressed in *Pdcd5::*d*Luc* driven bioluminescence (Figure 5). The few cultures expressing ultradian rhythms in bioluminescence in the negative controls (NT and NL; 9 and 10 rhythmic cultures, respectively) and non-synchronised cultures (Ns, 4 rhythmic cultures) exhibited periods of 4.1 h (±0.2 h; SD), 4.4 h (±0.5 h) and 5.3 h (±1.8 h), respectively (Figure 6). In cultures that were grown and measured in standard glucose concentrations, the ultradian period in *Pdcd5::*d*Luc* on average was 7.9 h (*±*1.9 h; S/S). In cultures that had been placed in reduced glucose concentrations only at pre-synchronisation, an average ultradian period of 8.2 h (±2.7 h; Figure 6) was observed whereas an ultradian period of 6.9 h (±1.1 h; Figure 6) was observed in cultures that were placed in reduced glucose concentrations at both pre- and post-synchronisation. Ultradian periods observed from cultures in standard glucose concentrations (S/S) and reduced glucose only at pre-synchronisation (R/S) were significantly longer than the negative controls (NT and NL; Tukey’s HSD test, *p* < 0.01, see Supplementary Table S2). The ultradian period for cultures in reduced glucose only at pre-synchronisation were also significantly longer than that of cultures in reduced glucose concentrations both at pre- and post-synchronisation (R/R; Tukey’s HSD test, *p* < 0.002), depicting much shorter average ultradian periods under reduced glucose concentrations throughout. When compared between different glucose concentrations within the R/S and S/S groups, no significant differences in period length were found (Tukey’s HSD test, *p* > 0.05).

**FIGURE 7:**
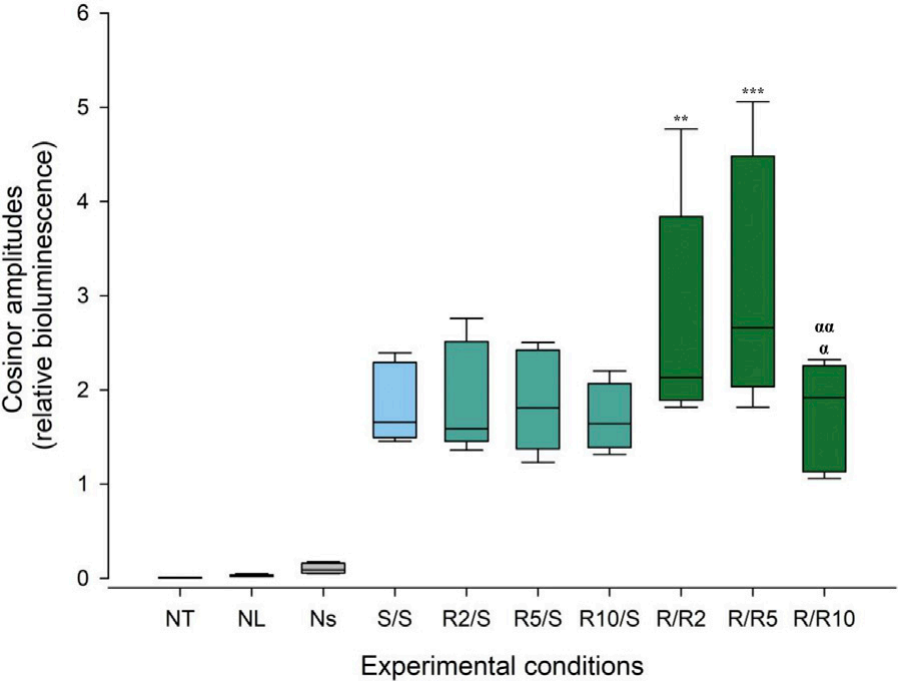
Cosinor amplitudes of ultradian rhythms of *Pdcd5::*d*Luc* bioluminescence under different glucose conditions. Box plot representing the spread of the cosinor amplitude values across experimental conditions and negative controls. Comparison between experimental conditions against the negative controls (NT and NL) and comparison of cosinor amplitudes between glucose concentrations were performed using two-way ANOVA. **p < 0.01 and ***p < 0.001 against the standard concentration (S/S), ^α^ *p* < 0.0005 against R/R2, ^αα^ *p* < 0.0001 against R/R5.

We next looked at the amplitude of the cosine fits as a measure of prominence of ultradian rhythmicity. Within the negative controls, cosinor amplitudes were 0.001 counts/sec (±0.002), 0.009 counts/sec (±0.012) and 0.038 counts/sec (±0.047) for NT, NL, and NS, respectively (Figure 7, Supplementary Table S3). Amplitudes in all experimental groups were significantly higher than the negative control conditions (Figure 7, Supplementary Table S2, two-way ANOVA, Tukey’s HSD test, *p* < 0.05, see Supplementary Table S4, S5). Crucially, amplitudes of ultradian rhythmicity in *Pdcd5::*d*Luc*-driven bioluminescence in the experimental groups that were measured under conditions of normal glucose concentration (S/S and R/S) were significantly lower than the amplitudes measured under reduced glucose concentration at 2 mM (R/R2) and 5 mM (R/R5) (two-way ANOVA, Tukey’s HSD test, *p* < 0.001). Cosinor amplitudes of ultradian rhythms from cells that were incubated at 10 mM glucose pre- and post-synchronisation exhibited an amplitude that was not significantly different from the normal glucose concentration (S/S and R/S).

## 4 Discussion

Our novel approach in pre-adipocyte cells exposed substantial intrinsically-driven ultradian rhythmicity in *Pdcd5* promotor activity *in vitro*. Ultradian rhythmicity was apparent in >80% of cultures in which the circadian clocks were synchronised, which is in line with other observations that ultradian rhythms are synchronised to circadian rhythms (Gerkema et al., 1993; Psomas et al., 2023). Crucially, we show that the ultradian amplitude is enhanced, and the ultradian period is shortened, in cells cultured at low glucose concentrations. The glucose levels tested were below levels that are considered euglycemic in mice (approximately <10 mM; Berglund et al., 2008; Ayala et al., 2010). These observations support our hypothesis that an unfavourable energy balance enhances ultradian rhythmicity *in vitro* and suggest that ultradian rhythmicity is linked to metabolic homeostasis at a cellular level.

We observed that even under normal glucose concentrations, most cultures exhibited ultradian rhythms in *Pdcd5*-driven bioluminescence. The recorded *Pdcd5* driven bioluminescence signal is an integration of signals originating from all cells in the culture combined, and ultradian rhythmicity in this signal was only evident after circadian rhythms within the cultures were synchronised using dexamethasone (Balsalobre et al., 1998; Balsalobre et al., 2000). When placing cultures in the lumicycle, the sealed system provided a challenge which was addressed by performing longevity testing on the cultures we place in the lumicycle and have ensured that the results presented are based on living cultured cells. Previous reports on ultradian feeding behaviour in the vole also reported that the phase of behavioural ultradian rhythms were synchronised by circadian rhythms (Gerkema et al., 1993; Psomas et al., 2023), and it could be that this is also the case for ultradian rhythms in *Pdcd5* expression *in vitro*.

Our analysis also identified some low-amplitude, short-period ultradian rhythms in a minority of our negative control cultures. These control conditions either do not contain the reporter construct or the substrate luciferin to measure bioluminescence and these signals therefore do not originate from our construct and were excluded because their amplitude and period fell below that observed in the experimental conditions. These signals are not considered to be ultradian rhythms and may be attributed to several technical aspects such as autofluorescence, intrinsic oscillatory bias of photon detection in the instrumentation, or other noise originating from the equipment. Our experimentally-induced amplitude enhancement of ultradian rhythms in *Pdcd5* expression *in vitro* using low glucose concentrations are in line with the enhancement of ultradian behavioural rhythms seen during negative energy balance (van Oort et al., 2007; van Beest et al., 2020; van Rosmalen and Hut, 2021). This finding suggests that the re-balancing of ultradian and circadian metabolic rhythms in response to a metabolic challenge already occurs at a cellular level. Within this project, ultradian rhythm amplitudes were observed to be significantly enhanced in cultures that were acutely cultured at glucose concentrations of 2 and 5 mM, but not at 10 mM. The pre-adipocyte cells were originally isolated from mice, and blood glucose levels of ∼10 mM are considered euglycemic in mice, whereas concentrations <10 mM are considered a hypoglycaemic state (Ayala et al., 2010). Our results thus suggest that the increased prominence of ultradian rhythmicity *in vitro* could be related to acute cellular hypoglycaemic conditions.

Besides an increase in ultradian amplitude in *Pdcd5* expression, acute low glucose conditions also associated with shorter ultradian periods. It could be argued that the period shortening suggests that ultradian rhythms are not driven by a buffered, or compensated oscillator mechanism such as the molecular circadian clock (Takahashi, 2017), and indeed it has already been established that ultradian behavioural rhythms do not rely on the presence of the circadian clock (Gerkema and Daan, 1990; Schwartz and Zimmerman, 1991; Vitaterna et al., 1994; Bunger et al., 2000; Zheng et al., 2001; Prendergast et al., 2012; Prendergast and Zucker, 2016; Goh et al., 2019; Aviram et al., 2021; Ballance and Zhu, 2021). Another reason for this period shortening could be that low glucose availability directly modulates the physiology that drives ultradian rhythms, such as cyclical, or autoregulatory processes in cellular metabolism. The latter hypothesis is tempting given that ultradian rhythms are often observed in systemic and cellular metabolism (Brodsky, 2014; Aviram et al., 2021; Ghenim et al., 2021; Yang et al., 2022; Psomas et al., 2023) and provides a new focus to the search for ultradian oscillatory mechanisms.

Our choice for using *Pdcd5* as a model to investigate ultradian rhythms in gene expression was based on our observation of robust *Pdcd5* ultradian mRNA expression patterns *in vitro* (van der Veen and Gerkema, 2017). The function of *Pdcd5* ranges from tumour suppression to positive regulation of apoptosis, specifically binding to tumour suppressors, such as p53 (Liu et al., 1999; Xu et al., 2012; Li et al., 2013). Moreover, *Pdcd5* has been associated with arresting the cell cycle (Li et al., 2017), and we previously saw that ultradian gene expression was enriched for genes associated with the cell cycle (van der Veen and Gerkema, 2017). Apoptosis has also been shown to be modulated by circadian clock genes (Liu et al., 2021), and therefore co-expression of these biological rhythms with different cell cycle lengths indicates the importance in intricately regulating this system.

A link between ultradian rhythms and metabolic homeostasis has long been hypothesised (Aschoff and Meyer-Lohmann, 1954; Aschoff, 1960; Aschoff and Gerkema, 1985), and more recently evidence for a close relationship between ultradian rhythmicity and metabolism has been accumulating. Here, we developed a novel *in vitro* model for cellular ultradian rhythmicity and showed that reduced energy availability enhanced ultradian rhythmicity at a cellular level. Our findings confirm the intimate link between cellular metabolic homeostasis and ultradian rhythmicity and suggest that a reduction in energy availability directly enhances ultradian amplitude. Recent developments, in line with our results, also show a relationship between glutamine and ultradian rhythms *in vitro* providing further evidence of the interaction between metabolism (Yang et al., 2022). This presents an opportunity to investigate the role of ultradian rhythms in metabolism using glutamine and other energy sources. The dynamic interplay between glucose concentration and ultradian rhythms observed offer new insights into the functional significance of ultradian rhythmicity, suggesting these intrinsically driven biological rhythms serve metabolic homeostasis at a cellular level.

## Data Availability

The raw data supporting the conclusion of this article will be made available by the authors, without undue reservation.

## References

[B1] Asher G. Zhu B. (2023). ‘Beyond circadian rhythms: emerging roles of ultradian rhythms in control of liver functions’. Hepatology 77 (3), 1022–1035. 10.1002/hep.32580 35591797 PMC9674798

[B2] Aschoff J. Gerkema M. (1985). in Ultradian rhythms in physiology and behavior. Editors Schulz H. Lavie P. 1st edn. (Heidelberg: Springer Berlin).

[B3] Aschoff J. Meyer-Lohmann J. (1954). ‘[Burst sequence of locomotoric activity in rodents]’. Pflugers Arch. fur gesamte Physiol. Menschen Tiere 260 (1), 81–86. 10.1007/BF00363781 13236478

[B4] Aschoff J. (1960). ‘Exogenous and endogenous components in circadian rhythms’. Cold Spring Harb. symposia quantitative Biol. 25, 11–28. 10.1101/sqb.1960.025.01.004 13684695

[B5] Aviram R. Dandavate V. Manella G. Golik M. Asher G. (2021). ‘Ultradian rhythms of AKT phosphorylation and gene expression emerge in the absence of the circadian clock components Per1 and Per2’. PLoS Biol. 19 (12), e3001492. 10.1371/journal.pbio.3001492 34968386 PMC8718012

[B6] Ayala J. E. Samuel V. T. Morton G. J. Obici S. Croniger C. M. Shulman G. I. NIH Mouse Metabolic Phenotyping Center Consortium (2010). ‘Standard operating procedures for describing and performing metabolic tests of glucose homeostasis in mice.’. Dis. models Mech. 3 (9–10), 525–534. 10.1242/dmm.006239 PMC2938392 20713647

[B7] Ballance H. Zhu B. (2021). Revealing the hidden reality of the mammalian 12-h ultradian rhythms’, *Cellular and Molecular Life Sciences* . Springer Science and Business Media Deutschland GmbH, 3127–3140. 10.1007/s00018-020-03730-5 ‘ PMC8407301 33449146

[B8] Balsalobre A. Brown S. A. Marcacci L. Tronche F. Kellendonk C. Reichardt H. M. (2000). ‘Resetting of circadian time in peripheral tissues by glucocorticoid signaling.’. Sci. (New York, N.Y.) 289 (5488), 2344–2347. 10.1126/science.289.5488.2344 11009419

[B9] Balsalobre A. Damiola F. Schibler U. (1998). ‘A serum shock induces circadian gene expression in mammalian tissue culture cells.’. Cell 93 (6), 929–937. 10.1016/s0092-8674(00)81199-x 9635423

[B10] Berglund E. D. Li C. Y. Poffenberger G. Ayala J. E. Fueger P. T. Willis S. E. (2008). ‘Glucose metabolism *in vivo* in four commonly used inbred mouse strains’. Diabetes 57 (7), 1790–1799. 10.2337/db07-1615 18398139 PMC2453626

[B11] Bloch G. Barnes B. M. Gerkema M. P. Helm B. (2013). ‘Animal activity around the clock with no overt circadian rhythms: patterns, mechanisms and adaptive value’. Proc. R. Soc. B Biol. Sci. 280 (1765), 20130019. 10.1098/rspb.2013.0019 PMC3712434 23825202

[B12] Blum I. D. Zhu L. Moquin L. Kokoeva M. V. Gratton A. Giros B. (2014). ‘A highly tunable dopaminergic oscillator generates ultradian rhythms of behavioral arousal’. eLife 3, e05105. 10.7554/eLife.05105 25546305 PMC4337656

[B13] Brodsky V. Y. (2014). ‘Circahoralian (Ultradian) metabolic rhythms’. Biochem. Mosc. 79 (6), 483–495. 10.1134/S0006297914060017 25100006

[B14] Bunger M. K. Wilsbacher L. D. Moran S. M. Clendenin C. Radcliffe L. A. Hogenesch J. B. (2000). ‘Mop3 is an essential component of the master circadian pacemaker in mammals’. Cell 103 (7), 1009–1017. 10.1016/S0092-8674(00)00205-1 11163178 PMC3779439

[B15] Diatroptov M. E. Diatroptova M. A. Aleksankina V. V. Kosyreva A. M. (2020). ‘Ultradian biorhythms of C57bl/6 mice body temperature under constant illumination or during natural day-night cycle’. Bull. Exp. Biol. Med. 169 (3), 388–392. 10.1007/s10517-020-04893-8 32748138

[B16] Diatroptov M. E. Rutovskaya M. V. Kuznetsova E. V. Diatroptova M. A. Kosyreva A. M. Dzhalilova D. S. (2019). ‘Infradian and ultradian rhythms of body temperature resumption during hibernation’. Bull. Exp. Biol. Med. 168 (2), 291–294. 10.1007/s10517-019-04693-9 31782004

[B17] Dowse H. Umemori J. Koide T. (2010). ‘Ultradian components in the locomotor activity rhythms of the genetically normal mouse, *Mus musculus*.’. J. Exp. Biol. 213 (10), 1788–1795. 10.1242/jeb.038877 20435830

[B18] Droste S. K. de Groote L. Atkinson H. C. Lightman S. L. Reul J. M. H. M. Linthorst A. C. E. (2008). ‘Corticosterone levels in the brain show a distinct ultradian rhythm but a delayed response to forced swim stress.’. Endocrinology 149 (7), 3244–3253. 10.1210/en.2008-0103 18356272

[B19] Dull T. Zufferey R. Kelly M. Mandel R. J. Nguyen M. Trono D. (1998). A third-generation Lentivirus vector with a conditional packaging system. J. VIROLOGY 72 (11), 8463–8471. 10.1128/JVI.72.11.8463-8471.1998 9765382 PMC110254

[B20] Enright J. T. (1989). ‘The parallactic view, statistical testing, and circular reasoning.’. J. Biol. rhythms 4 (2), 183–192. 10.1177/074873048900400214 2519595

[B21] Flynn B. P. Conway-Campbell B. L. Lightman S. L. (2018). ‘The emerging importance of ultradian glucocorticoid rhythms within metabolic pathology.’. Ann. d’endocrinologie 79 (3), 112–114. 10.1016/j.ando.2018.03.003 PMC5984398 29627070

[B22] Gerkema M. P. Daan S. Wilbrink M. Hop M. W. van der Leest F. (1993). ‘Phase control of ultradian feeding rhythms in the common vole (*Microtus arvalis*): the roles of light and the circadian system.’. J. Biol. rhythms 8 (2), 151–171. 10.1177/074873049300800205 8369551

[B23] Gerkema M. P. Daan S. (1990). ‘Differential elimination of circadian and ultradian rhythmicity by hypothalamic lesions in the common vole, *Microtus arvalis*’. J. Biol. Rhythms 5 (2), 81–95. 10.1177/074873049000500201 2133128

[B24] Gerkema M. P. van der Leest F. (1991). ‘Ongoing ultradian activity rhythms in the common vole, *Microtus arvalis*, during deprivations of food, water and rest’. J. Comp. Physiology A 168 (5), 591–597. 10.1007/BF00215081 1920159

[B25] Ghenim L. Allier C. Obeid P. Hervé L. Fortin J.-Y. Balakirev M. (2021). ‘A new ultradian rhythm in mammalian cell dry mass observed by holography’. Sci. Rep. 11 (1), 1290. 10.1038/s41598-020-79661-9 33446678 PMC7809366

[B26] Goh G. H. Maloney S. K. Mark P. J. Blache D. (2019). ‘Episodic ultradian events-ultradian rhythms.’. Biology 8 (1), 15. 10.3390/biology8010015 30875767 PMC6466064

[B27] Hughes M. E. DiTacchio L. Hayes K. R. Vollmers C. Pulivarthy S. Baggs J. E. (2009). ‘Harmonics of circadian gene transcription in mammals.’. PLoS Genet. 5 (4), e1000442. 10.1371/journal.pgen.1000442 19343201 PMC2654964

[B28] Kalafatakis K. Russell G. M. Harmer C. J. Munafo M. R. Marchant N. Wilson A. (2018). ‘Ultradian rhythmicity of plasma cortisol is necessary for normal emotional and cognitive responses in man’. Proc. Natl. Acad. Sci. 115 (17), E4091–E4100. 10.1073/pnas.1714239115 29632168 PMC5924881

[B29] Leclerc G. M. Boockfor F. R. Faught W. J. Frawley L. S. (2000). ‘Development of a destabilized firefly luciferase enzyme for measurement of gene expression.’. Biotechniques. 29 (3), 590–591. 10.2144/00293rr02 10997273

[B30] Li P. Fei H. Wang L. Xu H. Zhang H. Zheng L. (2017). PDCD5 regulates cell proliferation, cell cycle progression and apoptosis. Oncol. Lett. Prepr. 15, 1177–1183. 10.3892/ol.2017.7401 PMC5780840 29403562

[B31] Li Y. Zhou G. La L. Chi X. Cao Y. Liu J. (2013). ‘Transgenic human programmed cell death 5 expression in mice suppresses skin cancer development by enhancing apoptosis. Life Sci. 92 (24–26), 1208–1214. 10.1016/j.lfs.2013.05.005 23688867

[B32] Lightman S. L. Wiles C. C. Atkinson H. C. Henley D. E. Russell G. M. Leendertz J. A. (2008). ‘The significance of glucocorticoid pulsatility’. Eur. J. Pharmacol. 583 (2–3), 255–262. 10.1016/j.ejphar.2007.11.073 18339373

[B33] Lindsley G. Dowse H. B. Burgoon P. W. Kolka M. A. Stephenson L. A. (1999). ‘A persistent circhoral ultradian rhythm is identified in human core temperature.’. Chronobiology Int. 16 (1), 69–78. 10.3109/07420529908998713 10023577

[B34] Liu H. Wang Y. Zhang Y. Song Q. Di C. Chen G. (1999). ‘TFAR19,a novel apoptosis-related gene cloned from human leukemia cell line TF-1, could enhance apoptosis of some tumor cells induced by growth factor withdrawal’. Biochem. Biophysical Res. Commun. 254 (1), 203–210. 10.1006/bbrc.1998.9893 9920759

[B35] Liu S. Cheng Y. Wang S. Liu H. (2021). ‘Circadian clock genes modulate immune, cell cycle and apoptosis in the diagnosis and prognosis of pan-renal cell carcinoma’. Front. Mol. Biosci. 8, 747629. 10.3389/fmolb.2021.747629 34977153 PMC8717949

[B36] Morris M. Yamazaki S. Stefanovska A. (2022). ‘Multiscale time-resolved analysis reveals remaining behavioral rhythms in mice without canonical circadian clocks’. J. Biol. Rhythms 37 (3), 310–328. 10.1177/07487304221087065 35575430 PMC9160956

[B37] Prendergast B. J. Cisse Y. M. Cable E. J. Zucker I. (2012). ‘Dissociation of ultradian and circadian phenotypes in female and male Siberian hamsters.’. J. Biol. rhythms 27 (4), 287–298. 10.1177/0748730412448618 22855573 PMC3965331

[B38] Prendergast B. J. Zucker I. (2016). ‘Ultradian rhythms in mammalian physiology and behavior’. Curr. Opin. Neurobiol. 40, 150–154. 10.1016/j.conb.2016.07.011 27568859

[B39] Psomas A. Chowdhury N. R. Middleton B. Winsky-Sommerer R. Skene D. J. Gerkema M. P. (2023). ‘Co-expression of diurnal and ultradian rhythms in the plasma metabolome of common voles (*Microtus arvalis*)’. FASEB J. 37 (4), e22827. 10.1096/fj.202201585R 36856610 PMC11977602

[B40] Schwartz W. J. Zimmerman P. (1991). ‘Lesions of the suprachiasmatic nucleus disrupt circadian locomotor rhythms in the mouse’. Physiology Behav. 49 (6), 1283–1287. 10.1016/0031-9384(91)90364-T 1896511

[B41] Scott M. R. Zong W. Ketchesin K. D. Seney M. L. Tseng G. C. Zhu B. (2023). ‘Twelve-hour rhythms in transcript expression within the human dorsolateral prefrontal cortex are altered in schizophrenia’. PLOS Biol. 21 (1), e3001688. 10.1371/journal.pbio.3001688 36693045 PMC9873190

[B42] Szymanski J. S. (1920). ‘Aktivität und ruhe bei tieren und menschen’. Z. Allg. Physiol. 18, 105–162.

[B43] Takahashi J. S. (2017). ‘Transcriptional architecture of the mammalian circadian clock’. Nat. Rev. Genet. 18 (3), 164–179. 10.1038/nrg.2016.150 27990019 PMC5501165

[B44] Thiel A. Giroud S. Hertel A. G. Friebe A. Devineau O. Fuchs B. (2022). ‘Seasonality in biological rhythms in scandinavian brown bears’. Front. Mater. 13, 785706. 10.3389/fphys.2022.785706 PMC9118031 35600291

[B45] van Beest F. Beumer L. Chimienti M. Desforges J.-P. Huffeldt N. Pedersen S. (2020). ‘Environmental conditions alter behavioural organization and rhythmicity of a large Arctic ruminant across the annual cycle’. R. Soc. Open Sci. 7, 201614. 10.1098/rsos.201614 33204486 PMC7657931

[B46] Van Cauter E. (1990). ‘Diurnal and ultradian rhythms in human endocrine function: A minireview.’. Hormone Res. 34 (2), 45–53. 10.1159/000181794 1965834

[B47] van der Veen D. R. Gerkema M. P. (2017). ‘Unmasking ultradian rhythms in gene expression’. FASEB J. 31 (2), 743–750. 10.1096/fj.201600872R 27871062 PMC5240665

[B48] van der Veen D. R. Minh N. L. Gos P. Arneric M. Gerkema M. P. Schibler U. (2006). ‘Impact of behavior on central and peripheral circadian clocks in the common vole *Microtus arvalis*, a mammal with ultradian rhythms.’. Proc. Natl. Acad. Sci. U. S. A. 103 (9), 3393–3398. 10.1073/pnas.0507825103 16481616 PMC1413878

[B49] van der Veen D. R. Saaltink D.-J. Gerkema M. P. (2011). ‘Behavioral responses to combinations of timed light, food availability, and ultradian rhythms in the common vole (*Microtus arvalis*)’. Chronobiology Int. 28 (7), 563–571. 10.3109/07420528.2011.591953 21790327

[B50] van Oort B. E. H. Tyler N. J. C. Gerkema M. P. Folkow L. Stokkan K.-A. (2007). ‘Where clocks are redundant: weak circadian mechanisms in reindeer living under polar photic conditions’. Naturwissenschaften 94 (3), 183–194. 10.1007/s00114-006-0174-2 17131139

[B51] van Rosmalen L. Hut R. A. (2021). ‘Negative energy balance enhances ultradian rhythmicity in spring-programmed voles.’. J. Biol. rhythms 36, 359–368. 10.1177/07487304211005640 33878968 PMC8276337

[B52] Vitaterna M. H. King D. P. Chang A.-M. Kornhauser J. M. Lowrey P. L. McDonald J. D. (1994). ‘Mutagenesis and mapping of a mouse gene, *clock*, essential for circadian behavior’. Science 264 (5159), 719–725. 10.1126/science.8171325 8171325 PMC3839659

[B53] Waite E. J. Mckenna M. Kershaw Y. Walker J. J. Cho K. Piggins H. D. (2012). ‘Ultradian corticosterone secretion is maintained in the absence of circadian cues’. Eur. J. Neurosci. 36 (8), 3142–3150. 10.1111/j.1460-9568.2012.08213.x 22823558

[B54] Wu Y. E. Enoki R. Oda Y. Huang Z. L. Honma K. I. Honma S. (2018). ‘Ultradian calcium rhythms in the paraventricular nucleus and subparaventricular zone in the hypothalamus’. Proc. Natl. Acad. Sci. U. S. A. 115 (40), E9469–E9478. 10.1073/pnas.1804300115 30228120 PMC6176559

[B55] Xu L. Hu J. Zhao Y. Hu J. Xiao J. Wang Y. (2012). ‘PDCD5 interacts with p53 and functions as a positive regulator in the p53 pathway’. Apoptosis 17 (11), 1235–1245. 10.1007/s10495-012-0754-x 22914926

[B56] Yang S. Yamazaki S. Cox K. H. Huang Y.-L. Miller E. W. Takahashi J. S. (2022). ‘Coupling-dependent metabolic ultradian rhythms in confluent cells’. Proc. Natl. Acad. Sci. U. S. A. 119. 10.1073/pnas.2211142119 PMC9659342 36322771

[B57] Zheng B. Albrecht U. Kaasik K. Sage M. Lu W. Vaishnav S. (2001). ‘Nonredundant roles of the mPer1 and mPer2 genes in the mammalian circadian clock’. Cell 105 (5), 683–694. 10.1016/S0092-8674(01)00380-4 11389837

[B58] Zhu B. Zhang Q. Pan Y. Mace E. M. York B. Antoulas A. C. (2017). ‘A cell-autonomous mammalian 12 hr clock coordinates metabolic and stress rhythms’. Cell Metab. 25 (6), 1305–1319. 10.1016/j.cmet.2017.05.004 28591634 PMC5526350

