# Supplementary material for: Reduced glucose concentration enhances ultradian rhythms in Pdcd5 promoter activity in vitro
Source: Front Physiol. 2023 Oct 11;14:1244497. doi: 10.3389/fphys.2023.1244497 (PMC10613464; doi:10.3389/fphys.2023.1244497)
Supplement: Supplementary file 1 [file DataSheet1.PDF]

*Supplementary Material*

# Reduced glucose concentration enhances ultradian rhythms in *Pdcd5* expression *in vitro*

**Isaiah J. Ting[1], Andreas Psomas[1], Debra J. Skene[1], Daan R. van der Veen**[1*]

[1]Chronobiology Section, Faculty of Health and Medical Sciences, University of Surrey, Guildford, UK.

*** Correspondence:**
Daan R van der Veen
d.vanderveen@surrey.ac.uk

Groups: **NT**: Non-transduced cultures; **NL**: Non-luciferin cultures; **Ns**: Non-synchronised cultures; **S/S**: Standard glucose in cultures pre-/post-synchronisation; **R/S**: Reduced glucose in cultures only pre-synchronisation, at different glucose concentrations of 2, 5 and 10 mM; **R/R**: Reduced glucose in cultures pre-/post-synchronisation, at different glucose concentrations of 2, 5 and 10 mM.

**Supplemental Table 1**

*P-values from chi-square analysis performed comparing the number of cultures (technical replicates) that were rhythmic vs. non-rhythmic, determined by cosinor analysis. Conditions with reduced glucose concentrations were also averaged and used to compare against the negative controls, indicated by [a] next to the condition.*

| Condition | NT | NL | Ns | S/S | R/S[a] | R/R[a] |
|---|---|---|---|---|---|---|
| **NT** | - | 0.846 | 0.0226 | < 0.0001 | < 0.0001 | < 0.0001 |
| **NL** | | - | 0.0740 | < 0.0001 | < 0.0001 | < 0.0001 |
| **Ns** | | | - | < 0.0001 | < 0.0001 | < 0.0001 |
| **S/S** | | | | - | 0.868 | 0.281 |
| **R/S[a]** | | | | | - | 0.329 |
| **R/R[a]** | | | | | | - |

**Supplemental Table 2**

*P-values from multiple comparison analysis, Tukey's HSD test, performed for the comparison of ultradian periods across the experimental conditions, determined by cosinor analysis. Conditions with reduced glucose concentrations were averaged and used to compare against the negative controls, indicated by [a] next to the condition.*

| Condition | NT | NL | Ns | S/S | R/S[a] | R/R[a] |
|---|---|---|---|---|---|---|
| **NT** | - | 0.9999 | 0.8346 | <0.0001 | <0.0001 | <0.0001 |
| **NL** | | - | 0.9368 | <0.0001 | <0.0001 | 0.0015 |
| **Ns** | | | - | 0.0289 | 0.0034 | 0.2528 |
| **S/S** | | | | - | 0.9764 | 0.3429 |
| **R/S[a]** | | | | | - | 0.0014 |
| **R/R[a]** | | | | | | - |

**Supplemental Table 3**

*Mean cosinor amplitudes of conditions with varying glucose concentrations tested using one-way ANOVA with Tukey's HSD test against the non-luciferin (NL) negative control.*

| Condition | Mean cosinor amplitude (counts/sec ± SD) | Amplitude tested against NL (P value, N) |
|---|---|---|
| **NT** | 0.001 ± 0.002 | - |
| **NL** | 0.009 ± 0.012 | - |
| **Ns** | 0.038 ± 0.047 | - |
| **S/S** | 1.82 ± 0.37 | - |
| **R2/S** | 1.87 ± 0.53 | P < 0.001, N = 12 |
| **R5/S** | 1.86 ± 0.47 | P < 0.001, N = 12 |
| **R10/S** | 1.71 ± 0.32 | P < 0.001, N = 12 |
| **R/R 2** | 2.72 ± 1.08 | P < 0.001, N = 12 |
| **R/R 5** | 3.10 ± 1.18 | P < 0.001, N = 12 |
| **R/R 10** | 1.77 ± 0.51 | P < 0.001, N = 12 |

## Supplemental Table 4.

*Two-way ANOVA comparing cosinor amplitudes within the different concentrations of reduced glucose concentrations at both pre-/post-synchronisations condition (R/R). Levels were the (1) effect of synchronising culture and (2) at different glucose concentrations (GC; S50: 50 mM glucose, R2/5/10: 2 mM, 5 mM, and 10 mM).*

| Source of variation | SS | Df | MS | F | P-value |
|---|---|---|---|---|---|
| **C** | 3.46 | 1 | 3.46 | F (1, 5) = 11.87 | 0.0183 |
| **GC** | 4.668 | 3 | 1.556 | F (3, 15) = 4.334 | 0.0218 |
| **C * GC** | 3.302 | 3 | 1.101 | F (3, 15) = 12.75 | 0.0002 |
| **Residual** | 1.295 | 15 | 0.08635 | - | - |

**C: Condition (R/R); GC: Glucose concentrations**

## Supplemental Table 5.

*Two-way ANOVA multiple comparison tests for cosinor amplitude. Tukey's HSD test was applied to the cosinor amplitudes across experimental conditions and P-values recorded for each comparison performed.*

| Condition | S/S | R2/S | R5/S | R10/S | R/R2 | R/R5 | R/R10 |
|---|---|---|---|---|---|---|---|
| **S/S** | - | 0.9926 | 0.9955 | 0.902 | 0.0005 | <0.0001 | 0.9887 |
| **R2/S** | 0.9926 | - | 0.9999 | 0.7756 | | | |
| **R5/S** | 0.9955 | 0.9999 | - | 0.7978 | | | |
| **R10/S** | 0.902 | 0.7756 | 0.7978 | - | | | |
| **R/R2** | 0.0005 | | | | - | 0.1551 | 0.0003 |
| **R/R5** | <0.0001 | | | | 0.1551 | - | <0.0001 |
| **R/R10** | 0.9887 | | | | 0.0003 | <0.0001 | - |
